# Contrast-enhanced MRI of the knee in asymptomatic pediatric controls compared to juvenile idiopathic arthritis patients to validate synovitis scores

**DOI:** 10.1186/1546-0096-12-S1-P14

**Published:** 2014-09-17

**Authors:** Charlotte M Nusman, Robert Hemke, Marc Benninga, Angelika Kindermann, Marion van Rossum, Taco Kuijpers, Mario Maas

**Affiliations:** 1 Radiology, Academic Medical Center, Netherlands; 2 Pediatric Hematology, Immunology, Rheumatology and Infectious Disease, Netherlands; 3 Pediatric Gastroenterology, Emma Children's Hospital AMC, Netherlands; 4 Pediatrics, Emma Children's Hospital AMC, Netherlands; 5 Pediatric Rheumatology, Reade Institute, location Jan van Breemen, Amsterdam, Netherlands

## Introduction

The primary target of disease in juvenile idiopathic arthritis (JIA) is inflamed synovium, i.e. synovitis, which can be best visualized with magnetic resonance imaging (MRI) upon administration of intravenous (IV) contrast. Adequate differentiation between pathologic from physiologic extent of synovial enhancement has important implications for (dis)continuation of therapy.

## Objectives

To describe the appearance of the healthy knee on MRI after IV contrast and to compare the enhancing synovium in asymptomatic children to JIA patients.

## Methods

An axial fat-saturated T1-weighted MRI sequence of the knee of 25 asymptomatic controls and 25 JIA patients was collected, blinded and randomized. The asymptomatic controls were children who underwent MR enterography with IV contrast for unrelated diseases, had no (history of) joint complaints or signs of joint inflammation and gave permission for an additional sequence of the knee. JIA patients were age/sex-matched and divided in three clinical subgroups: new-active, relapse and inactive. Two readers independently scored synovial hypertrophy (SH) on a scale from 0-2 (none, 2-4mm, ≥4mm) at six locations, based on the Juvenile Arthritis MRI Scoring system (JAMRIS). Afterwards agreement on incongruent cases was obtained. Differences in SH score and (subgroups) of JIA patients were assessed.

## Results

Mean age of all subjects (42% female) was 13.5 years (SD 2.5). Contrast-enhanced thickened synovium was present upon imaging of the knee in 60% of the controls (total SH score range 1-3) and 76% of the patients. A significant difference (p=0.007) in the total SH score was found between controls and JIA children. SH score could differentiate controls from the clinically active JIA subgroup (p=0.002) but not from the clinically inactive JIA subgroup (p=0.303). Findings only observed in the asymptomatic group consisted of an diffuse ‘contrast-outfading’ pattern in 28% of the controls and ‘pseudo-enhancement’ of the cartilage at the posterior condyles only, that could be mistaken for true synovial inflammation.

## Conclusion

In asymptomatic children only very mild synovial enhancement was detected as well as two as yet undescribed findings representing potential pitfalls in the assessment of disease activity upon MRI of the knee. The existing, very reliable JAMRIS system for assessment of enhancing synovium can differentiate JIA patients from asymptomatic controls but only at group-level. For individual differentiation improved MRI scoring needs to be developed avoiding the measurement of synovial thickness scoring to further establish MRI as more accurate monitoring tool for JIA disease activity.

## Disclosure of interest

None declared.

